# Supplementary material for: Integrating genomic structural equation modeling and experimental validation to unravel the genetic basis of male genital lichen sclerosus
Source: Front Immunol. 2026 Jul 16;17:1857945. doi: 10.3389/fimmu.2026.1857945 (PMC13435250; doi:10.3389/fimmu.2026.1857945)
Supplement: Supplementary file 6 [file SupplementaryFile5.zip › result/fuma_PSYCH_factor_sCCA1_focus.chr14.chr14.29972145.32383265.pop1.pdf]

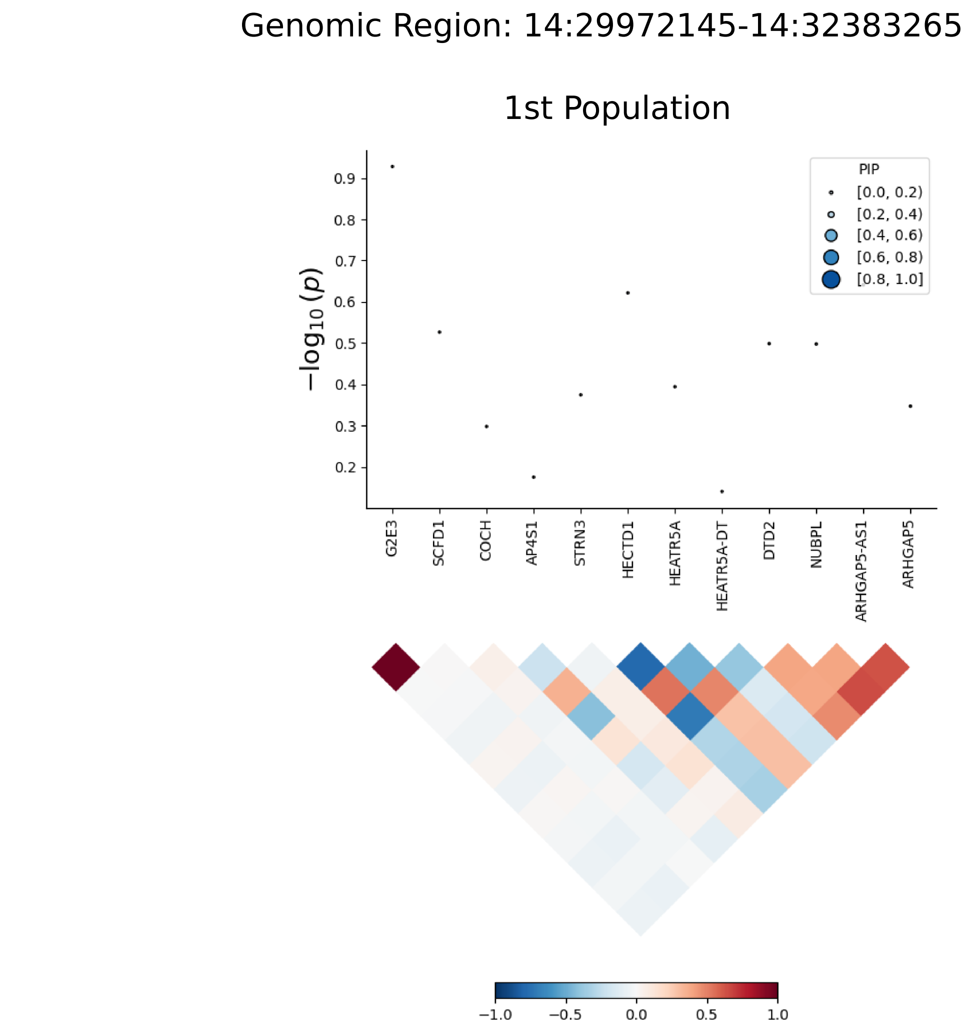
Genomic Region: 14:29972145-14:32383265
1st Population
PIP
[0.0, 0.2)
[0.2, 0.4)
[0.4, 0.6)
[0.6, 0.8)
[0.8, 1.0]
$-\log_{10}(p)$
0.9
0.8
0.7
0.6
0.5
0.4
0.3
0.2
G2E3
SCFD1
COCH
AP4S1
STRN3
HECTD1
HEATR5A
HEATR5A-DT
DTD2
NUBPL
ARHGAP5-AS1
ARHGAP5
−1.0
−0.5
0.0
0.5
1.0
